# Effects of exercise training on cardiac output in subjects with heart failure with preserved ejection fraction (HFpEF) — a protocol for a systematic review and meta-analysis

**DOI:** 10.1186/s13643-024-02529-w

**Published:** 2024-06-18

**Authors:** Raphael Schoch, Benedikt Gasser, Philippe Beck, Christian Appenzeller-Herzog, Arno Schmidt-Trucksäss

**Affiliations:** 1 Department of Sport, Exercise and Health, Division of Sport and Exercise Medicine, University of Basel, Grosse Allee 6, 4052 Basel, Switzerland; 2 University Medical Library, University of Basel, 4051 Basel, Switzerland; 3 Department of Clinical Research, University of Basel, 4031 Basel, Switzerland

**Keywords:** Heart failure with preserved ejection fraction (HFpEF), Cardiac output (CO), Exercise, Systematic review, Meta-analysis

## Abstract

**Background:**

Patients with heart failure with preserved ejection fraction (HFpEF) commonly experience exercise intolerance, resulting in reduced cardiorespiratory fitness. This is characterised by a decreased maximal oxygen uptake (V̇O_2peak_), which is determined by the product of cardiac output (CO) and arteriovenous oxygen difference (a-vDO_2_). While exercise training has been shown to improve V̇O_2peak_ in HFpEF patients, the effects on CO remain unclear. The aim of this study is to systematically review and analyse the current evidence on the effects of supervised exercise training on CO in patients with HFpEF.

**Methods:**

We will systematically search for literature describing the effects of supervised exercise training on CO in patients with HFpEF. All eligible studies published before 30 June 2023 in the following electronic databases will be included: MEDLINE (Ovid), Embase (Ovid), SPORTDiscus (EBSCOhost), and CENTRAL (Cochrane Library). Effect sizes will be extracted for CO before and after a supervised exercise training intervention at rest and maximal exercise. Mass of heterogeneity (*I*^2^) will be calculated, and either fixed-effect models or random-effect models will be used for meta-analysis. To detect a potential publication bias, funnel plot analyses will be performed.

**Discussion:**

While several studies have reported a positive effect of supervised exercise training on cardiorespiratory fitness, attempts to assess the underlying determinants of V̇O_2peak_, CO, and a-vDO_2_ are much scarcer, especially in patients with HFpEF. From a physiological perspective, measuring CO before and after supervised exercise training seems to be a reasonable way to accurately operationalise a potential improvement in cardiac function.

**Systematic review registration:**

PROSPERO CRD42022361485.

## Strengths and limitations of this study

- To the best of our knowledge, this is the first protocol planning to calculate the pooled effect estimate of supervised exercise training on CO in patients with HFpEF.
- By focusing on the specific physiological parameter CO, the systematic review will provide valuable insights into the impact of exercise training on cardiovascular function.
- A possible limitation will be the inclusion of studies written in English, French, and German only.
- A second possible limitation will be the possible heterogeneity in exercise training interventions. Variations in exercise duration, intensity, and frequency may have an impact on comparability and generalisability of the results.

## Introduction

Heart failure with preserved ejection fraction (HFpEF) is estimated to be prevalent in approximately 50% or more of individuals diagnosed with heart failure (HF) [1, 2]. It is defined as left ventricular ejection fraction > 50%, elevated levels of brain natriuretic peptides, objective evidence of structural heart disease or diastolic dysfunction [3], and associated with increased mortality, and health care expenditures [4]. A key characteristic of HFpEF is reduced cardiorespiratory fitness, defined as the maximal oxygen uptake (V̇O_2peak_) [4]. V̇O_2peak_ is the product of two main determinants: cardiac output (CO) and arteriovenous oxygen difference (a-vDO_2_). CO is the product of the heart rate (HR), the number of heart beats per minute, and the stroke volume (SV; the volume of blood pumped from the ventricle per beat). CO and a-vDO_2_ (and consequently V̇O_2peak_) are limited [5]. Nevertheless, in supervised exercise training studies, only the two markers V̇O_2peak_ and peak power output are usually reported [6], but neither CO nor a-vDO_2_.

While several studies have investigated the physiological mechanism underlying the reduced V̇O_2peak_ in patients with HF with reduced ejection fraction (HFrEF) [7–9], considerably, less is known about the mechanism in patients with HFpEF [10]. Although several studies have demonstrated that exercise training improves V̇O_2peak_ in patients with HFpEF [11–15], less is known about the effect of exercise training on CO in patients with HFpEF. Abudiab et al. showed that reduced cardiorespiratory fitness is associated with an insufficient CO in relation to metabolic demand [16]. In contrast, Haykowsky et al. reported that increased a-vDO_2_ is primarily contributing to improved V̇O_2peak_ in patients with HFpEF, while SV and CO were not significantly altered after 16 weeks of endurance training [17]. Similar to these results, Fu et al. demonstrated that improvement in V̇O2peak with high-intensity interval training was associated with an improved a-vDO2. However, maximal HR, SV index, and cardiac index remained unchanged after training [18].

On a general note, the improvement in exercise capacity is mainly operationalised with V̇O_2peak_, which does not provide clear indications of training effects on direct cardiac function like CO. As there is still a lack of understanding concerning adaption processes in patients with HFpEF, a thorough examination of the effect of supervised exercise training on CO at rest and maximal exercise is crucial and will assist the development of valuable therapeutic recommendations [19].

Furthermore, existing reviews have focused on parameters such as the ratio of peak early to late mitral inflow velocities (E/A ratio), the ratio of early diastolic mitral inflow to annular velocities (E/E’ ratio), or changes in left ventricular ejection fraction during resting conditions. These reviews and meta-analyses contributed significantly to the understanding of HFpEF pathophysiology [20–26].

However, our review aims to specifically examine the impact of exercise training on cardiac output at rest and during exercise in patients with HFpEF. By exploring the dynamic changes in cardiac output during exercise, our review seeks to elucidate novel insights into the pathophysiology of HFpEF. We believe that this approach will provide a comprehensive understanding of the physiological response to exercise in HFpEF, thereby advancing the current knowledge base in this field.

### Why it is important to do this review

Given the limited success of medical treatments in improving prognosis for patients with HFpEF [27–29] and the current therapeutic approaches primarily focused on relieving HF symptoms [19], it is crucial to better understand the effects of exercise training on V̇O_2peak_ and the underlying determinants CO and a-vDO_2_.

Therefore, to enhance the design of future studies and pinpoint therapeutic targets, a more comprehensive understanding of the basic mechanisms contributing to V̇O_2_ limitation in HFpEF is crucial [16].

This review is also important because it is still not clear which exercise intensity is best to improve the cardiorespiratory fitness in patients with HFpEF. Wisløff et al. (2007) demonstrated that high-intensity interval training (HIIT) was superior to moderate continuous training (MCT) in terms of improving cardiorespiratory fitness in heart failure patients [30]. Later, smaller studies were able to confirm these findings in patients with HFpEF [11, 12], but the recently published OptimEx study refuted previous findings and did not find superiority of HIIT compared to MCT in patients with HFpEF [31]. In order to determine which training protocol or exercise training is superior to another, an improvement in V̇O_2peak_ has often been considered as the crucial factor. It would, however, be beneficial to also obtain more information regarding the improvement of CO, as it is a critical determinant of exercise performance, and there is no consensus yet about the superior training protocol [11, 12, 31].

Furthermore, to the best of our knowledge, there is currently no systematic review and meta-analysis focusing on the effects of exercise training on CO in HFpEF. Therefore, reviewing and meta-analysing the literature to identify studies measuring CO in patients with HFpEF are a first step to determine whether exercise can improve CO, potentially assisting in the development of valid therapeutic recommendations for patients with HFpEF.

### Aim and review question

This study aims at systematically reviewing and meta-analysing the literature on effects of exercise training on CO in patients with HFpEF. As hypothesis with potential falsification, it shall be stated that an exercise training intervention as compared to standard of care has no effect on CO.

## Material and methods

This systematic review protocol is reported according to the guidelines of the Preferred Reporting Items for Systematic Reviews and Meta-Analyses Protocols (PRISMA-P) [32]. The protocol was submitted for registration in International Prospective Register of Systematic Reviews (PROSPERO) on 20 September 2022 and registered on 5 October 2022 (registration number CRD42022361485). The research question was formulated according to PICOS (Population, Intervention, Comparison, Outcome, Study type) framework (Table 1) with the aim of capturing the current evidence regarding the impact of exercise compared to standard care on the difference in CO from pre-to post-intervention.

**Table 1:** Population, intervention, comparison, outcome, and study design

Item	Specification
Population	Patients with HFpEF (any age)
Intervention	Supervised exercise training
Comparison	Standard care
Outcome	Prior-to-post-exercise training difference in CO at rest and maximal exercise
Study designs	Published RCTs only

*HFpEF* Heart failure with preserved ejection fraction, *CO* Cardiac output, *RCT* Randomised controlled trial

### Patient and public involvement

Patients or the public were not involved in the design, conduct, reporting, or dissemination plans of this research.

### Study eligibility criteria

Given the variability and challenge associated with the diagnosis of HFpEF [33], we have defined HFpEF by the following signs and symptoms: left ventricular ejection fraction > 50%, elevated levels of brain natriuretic peptides (BNP > 35 pg·ml−1 or NTproBNP > 125 pg·ml−1), objective evidence of structural heart disease, or diastolic dysfunction usually provided by echocardiography [3], without acute or severe coronary, valvular, or pulmonary disease that could mimic HF symptoms.

It is acknowledged that there might be diversity within the study populations concerning the definition of HFpEF. Therefore, we intend to provide a detailed description and discussion of the different definitions of HFpEF in the manuscript.

#### Inclusion criteria

- All studies investigating the effect of a supervised exercise training on CO in patients with HFpEF.
- All clinical studies, which were published until the date of the last search and have the design of a randomised controlled trial (with standard care applied to a control group)
- Studies measuring CO or SV at rest before and after a supervised exercise training
- All forms of supervised exercise training that can be considered having more than a single training session.

#### Exclusion criteria

- Studies reporting estimated CO
- Studies published in languages other than English, German, and French
- Study designs other than randomised controlled trial or non-original articles (for example editorials, letters, reviews), meta-analyses, case reports, and conference abstracts

### Information sources and search strategy

Search strategies were developed in collaboration with an information specialist (C. A.-H.) using the Peer Review of Electronic Search Strategies framework [34]. We will search MEDLINE (Ovid), Embase (Ovid), SPORTDiscus (EBSCOhost), and CENTRAL (Cochrane Library) for randomised controlled trials using database-specific subjects headings and text-word synonyms around the concepts HFpEF, exercise training, and CO. Nonhuman studies and conference abstracts will be excluded. The detailed search strings can be found in online supplemental document.

To complement the results of database searching, the bibliographic references and citing articles of all included articles will be collected from citationchaser, Scopus, and the Web of Science, deduplicated and screened for eligibility (backward and forward citation searching).

### Study records: data management, selection process, and data collection

Studies were imported into EndNote (version 21), to remove duplicates, and then entered into Rayyan [35] for the screening process. The titles and abstracts of retrieved records will be reviewed independently by two authors (P. B. and R. S.). Articles will be categorised as ‘include’,’exclude’, or ‘uncertain’ based on the prespecified eligibility criteria. For articles categorized as ‘include’ or ‘uncertain’, the full text will be retrieved and independently reviewed for eligibility by two authors (P. B. and R. S.). Any discrepancies during title/abstract or full-text screening will be resolved through discussion between the two screening authors. If no resolution can be found, a third party (B.) will make the final judgement. Data will be extracted from the full texts and entered into a standardised Excel form. One author will extract the data (B. G.), and a second author will independently check the extractions (R. S.). Any discrepancies will be resolved through discussion, involving a third party (A. S. T.) if necessary. Corresponding authors will be contacted twice via email in case of any missing or unclear data. Publications will be excluded from meta-analysis if there is no response or if the required data cannot be provided. The data to be extracted are shown in Table 2. If there are more than two assessment time points (beyond pre-post), the initial assessment and the one closest to the end of the intervention will be considered.

**Table 2:** Data that will be extracted from every study included in the review

No	Description
**1**	Authors and year publication
**2**	Country of study
**3**	Study population
**4**	Study population demographics (*n*, age, sex, BMI, cardiorespiratory fitness)
**5**	Study completion rate
**6** **7**	Details of exercise training intervention (type of training, number of training sessions, frequency, duration, and intensity of training)
**8**	CO at rest and maximal exercise prior to intervention
**9**	CO at rest and maximal exercise post intervention
**10**	CPET protocol and exercise exhaustion criteria

*BMI* Body mass index, *CO* Cardiac output, *CPET* Cardiopulmonary exercise testing

### Outcome and prioritisation

The primary outcome evaluated in this study is the difference in CO levels at rest and under peak power from before to after a supervised exercise training in subjects with HFpEF.

### Risks-of-bias (quality) assessment

The risk of bias at study level will be assessed using the RoB 2 tool by two authors independently (P. B. and R. S.) [36]. Discrepancies will be resolved through discussion (or with a third party, if necessary, AST).

### Data synthesis

Standard mean differences (SMD) at the study level will be calculated as the difference in mean change of CO before versus after exercise training or standard care for the control group. SMD will be interpreted as small *d* = 0.2, moderate *d* = 0.5, or large *d* ≥ 0.8. Furthermore, to detect the effect of exercise training, we will compare and meta-analyse the pre- versus post-differences in CO in the exercise training group and the control group by calculating a pooled SMD. Cochran’s Q statistic will be calculated, providing a measure of the variance between the SMD (with *p* < 0.05 indicating evidence for heterogeneity), while *I*^2^ provides a measure of the amount of variance between studies in terms of heterogeneity (with *I*^2^ > 50% indicating substantial heterogeneity) [37, 38]. Thereby, heterogeneity variance shall be estimated using restricted maximum likelihood method (REML). If study heterogeneity is substantial (*I*^2^ > 50%), random-effect models shall be used for meta-analysis of SMD (otherwise fixed-effect models). Statistical analyses will be conducted using Review Manager software (version 5.3, Copenhagen: The Nordic Cochrane Center, The Cochrane Collaboration 2014) and R (version 4.1.1) [39].

### Additional analysis

No additional analyses will be conducted.

### Meta-bias(es)

If 10 or more studies will be found, funnel plots according to Egger will be calculated to detect a potential publication bias [40].

### Certainty of cumulative evidence

The certainty of evidence will be evaluated with the Grading of Recommendations Assessment, Development and Evaluation system, which is a tool classifying evidence into one of four categories ranging from very low to high [41].

### Ethics and dissemination

The present work is a systematic review and meta-analysis protocol. No human participants will be involved; therefore, no ethics approval is required. It is planned to communicate the study results in a peer-reviewed journal and as a conference presentation.

## Conclusion

The aim of this systematic review and meta-analysis is to investigate the effects of supervised exercise training on CO in patients with HFpEF.

This research is of utmost importance because current exercise training interventions primarily focus on improving V̇O_2peak_, while the impact on CO, a critical determinant of exercise performance, remains largely unknown, and CO is crucial for understanding exercise intolerance in patients with HFpEF.

The systematic review and meta-analysis aims to address this knowledge gap and determine whether supervised exercise training has an effect on CO in patients with HFpEF in order to achieve a potential improvement of the therapeutic recommendations in the future.

## Data Availability

All data will be included in the final published systematic review and meta-analysis article.

## Abbreviations

a-vDO_2_: Arteriovenous oxygen difference
BMI: Body mass index
BNP: Brain natriuretic peptides
CO: Cardiac output
CPET: Cardiopulmonary exercise testing
HF: Heart failure
HFpEF: Heart failure with preserved ejection fraction
HFrEF: Heart failure with reduced ejection fraction
HIIT: High-intensity interval training
HR: Heart rate
MCT: Moderate continuous training
NTproBNP: N-terminal pro-B-type natriuretic peptide
PRISMA-P: Preferred Reporting Items for Systematic Reviews and Meta-Analyses Protocols
PROSPERO: International Prospective Register of Systematic Reviews
RCT: Randomised controlled trial
REML: Restricted maximum likelihood
SMD: Standard mean difference
SV: Stroke volume
V̇O_2peak_: Maximal oxygen uptake
